# Alleviating psychological distress associated with a positive cervical cancer screening result: a randomized control trial

**DOI:** 10.1186/s12905-021-01207-6

**Published:** 2021-02-12

**Authors:** Yukari Isaka, Ai Hori, Rie Tanaka, Masao Ichikawa

**Affiliations:** 1 Graduate School of Comprehensive Human Sciences, University of Tsukuba, 1-1-1 Tennodai, Tsukuba, Ibaraki 305-8577 Japan; 2 Faculty of Medicine, University of Tsukuba, 1-1-1 Tennodai, Tsukuba, Ibaraki 305-8577 Japan

**Keywords:** Cervical cancer, Cervical cancer screening, False-positive results, Psychological distress, Randomized control trial, Screening notification

## Abstract

**Background:**

The method of communicating a positive cancer screening result should seek to alleviate psychological distress associated with a positive result. We evaluated whether the provision of information through a leaflet would help reduce psychological distress in a randomized controlled trial.

**Methods:**

The participants were women aged 20–69 years who were about to undergo cervical cancer screening at health centers. Before the screening, they received hypothetical screening results, with a leaflet (intervention group, *n* = 493) or without it (control group, *n* = 479), randomly. Their psychological distress and intention to undergo further examination were then compared between the intervention and control groups.

**Results:**

After the intervention (providing a leaflet with hypothetical screening results), psychological distress appeared to be higher in the control group than in the intervention group among those who received a hypothetical positive screening result (odds ratio: 2.57, 95% confidence interval: 1.87–3.54), while 95% and 97% of those in the intervention and control groups, respectively, reported that they would undergo further examination.

**Conclusions:**

Information provision might help reduce psychological distress but not hinder further examination among women who screen positive for cervical cancer.

*Trial registration*: UMIN Clinical Trials Registry UMIN000029894. Date of Registration: November 2017.

## Background

Globally, cervical cancer is the fourth most common cancer among women, with about 567,000 incident cases and about 311,000 deaths in 2018 [1]. In Japan, cervical cancer is the fourth most common cancer among women [2, 3]. The mortality rate (per 100,000 population) of cervical cancer in Japan was 2.7 in 2018, which was higher than the rates in other developed countries: 1.9 in the United States, 1.7 in the United Kingdom, 1.7 in Australia, and 1.7 Canada [4]. To enable early detection and treatment of cervical cancer, the Japanese government recommends that women aged 20–69 years undergo cytology-based (Pap smear) screening every two years [5].

While cervical cancer screening is largely beneficial for women, a positive screening result might cause psychological burden [6]. In fact, our previous study involving women who had undergone cervical cancer screening found a greater level of psychological distress among those who had received a positive result than those who had not yet received a result. However, the positive predictive value of Pap smear screening was only 1.9% in the Japanese population (i.e., only 2 in 100 women with a positive screening result are finally diagnosed with cervical cancer) [6, 7]. Psychological distress associated with a positive result should not be overlooked because a positive result can influence the decision to undergo further examination, future screening for cervical cancer, and daily activities [8–12].

Psychological distress due to a positive screening result might be mitigated if women are made aware of the fact that this result does not necessarily indicate a cancer diagnosis. A randomized controlled trial conducted in the United Kingdom suggested that the provision of such information through a leaflet when notifying women of a positive result could help avoid adverse psychological effects [13]. Thus, in the United Kingdom, women participating in cervical cancer screening programs receive a leaflet that is tailored to explain every part of the process, such as screening, normal results, abnormal results, and treatment [14, 15]. In Japan, such information is not provided to women undergoing cervical cancer screening; they are only informed of a positive or negative result, generally through a notification letter. In the case of several local governments, the abbreviations of the cytological stages are used to indicate the cytology results. This information is intended not for women but for physicians who conduct further examinations.

Women who screen positive need to undergo further examination, and their intention to do so might be influenced by the level of psychological distress they experience [16, 17]. Therefore, the information provided during the cervical cancer screening process should serve the purpose of prompting women with a positive result to undergo further examination while alleviating psychological distress. To date, these two outcomes of information provision have not been investigated at the same time. Therefore, we previously developed a leaflet to achieve these two goals. In the present study, we conducted a questionnaire-based randomized controlled trial to evaluate whether the leaflet would help reduce psychological distress without affecting the intention to undergo further examination among women who were about to undergo population-based screening for cervical cancer.

## Methods

### Study design

This study was a simple randomized controlled trial that randomly assigned individual participants to the intervention and control groups and measured outcomes before and after the intervention. The trial was approved by the Research Ethics Committee of the Faculty of Medicine at the University of Tsukuba (No.1216) and registered in the University Hospital Medical Information Network Clinical Trials Registry (UMIN000029894). We also obtained official permission from Tsukuba City to conduct the trial at the City’s health centers. This article complies with the Consolidated Standards of Reporting Trials 2010 guideline [18, 19].

### Setting

We conducted this study in Tsukuba City, Ibaraki Prefecture, a semi-urban city located 50 km northeast of Tokyo, with a population of 237,000. In Japan, cancer screening is conducted as a health promotion service based on the Health Promotion Act. The local government introduced a population-based cervical cancer screening system in 1983. In 2001, the Research Group for Cancer Screening Guidelines commissioned by the Japanese government recommended the use of cytology-based screening for cervical cancer [5]. In Ibaraki Prefecture, women aged 20–69 years are invited to undergo cervical cancer screening every two years through a letter with a subsidized or free coupon. Those who undergo cervical cancer screening are informed of the results through a letter. In case of a positive result, the cytology result based on the Bethesda System (TBS; indicating the cytological stage) is included.

### Participants

The participants were women aged 20–69 years who were about to undergo cervical cancer screening at health centers in Tsukuba City from July 2017 to February 2018. Those who were able to read and write Japanese were eligible to participate in the study. We selected two health centers where a large number of women underwent cervical cancer screening. The participants were recruited by health centers’ public health nurses at the reception of screening venues. They explained the aim of the study in written materials and provided written informed consent if they agreed to participate.

The sample size required for this study was 814 participants (407 each for the intervention and control groups) to ensure an 80% power and 5% significance level, and with an assumption that the prevalence of psychological distress among women who received a positive screening result was 50%, based on our previous findings, and that the intervention could reduce it to 40% [6].

### Intervention

The provision of information through a leaflet was the intervention in this study. The leaflet contained basic information about cervical cancer and its screening, the advantages and disadvantages of screening (including information on false-positives, false-negatives, and overdiagnosis), purpose and validity of the screening/further examination, meaning and causes of a positive screening result, explanation of TBS [20], effectiveness of treatment, importance of undergoing further examination, and where to undergo further examination and how to make a reservation for it. We explicitly stated that a positive screening result does not indicate a cancer diagnosis.

To develop the leaflet, we first reviewed current screening result notification-related practices in Ibaraki Prefecture as well as the leaflets regarding cervical cancer screening developed by the National Cancer Center in the United States and the National Health Service in the United Kingdom [15]. Second, we conducted an individual interview and focus group discussion to understand exactly what information should be provided to women who undergo cervical cancer screening. For the interview, we invited 10 women aged 20–69 who visited the hospital to undergo further examination for cervical cancer in order to draft the leaflet. We then conducted two focus group discussions with a total of 12 women to make the necessary revisions of the drafted leaflet. For the focus group discussions, we recruited women aged 20–69 from the general population through advertisements. Finally, we checked the appropriateness of the leaflet with health professionals, including gynecologists.

### Procedure and randomization

The participants received self-administered questionnaires that consisted of baseline questions, a hypothetical notification letter of cervical cancer screening, the leaflet (only for the intervention group), and follow-up questions. A hypothetical notification letter indicated a positive or negative result. In the case of a positive result, the letter indicated a hypothetical cytological stage, one of the five TBS-based grades, or no stage. Thus, there were six patterns of positive results, plus a negative result.

In baseline questions, the participants rated their psychological distress. They were then instructed to read the hypothetical notification letter as if they were being informed of the actual screening results. Those in the intervention group read the leaflet after the notification letter. Finally, all the participants rated their psychological distress again in the follow-up questions.

The leaflet was randomly included in the questionnaires that were consecutively handed to the participants as they came to health centers for the screening. The questionnaires were handed only to those who agreed to participate. This was the procedure of random allocation of the participants to the intervention and control groups. Since the intervention (leaflet) was known to the participants but not to the authors or public health nurses who handed the questionnaires, the trial was single-blinded.

The participants were requested to answer and return the questionnaire while waiting to undergo screening. As an ethical consideration, all the participants were provided with the leaflet when they returned the questionnaire. They were also allowed to consult with public health nurses during the screening or later by phone or e-mail, in cases where they became too worried about the screening results because of this study and they wished to consult. The participants received actual screening results from health centers by mail about two weeks after the screening.

### Measurement

The primary outcome was cancer-related psychological distress. Psychological distress was measured using the Cancer Worry Scale [21, 22]. The Japanese version of this scale has been demonstrated to be valid in the context of breast cancer-related worry. In this study, we replaced “breast cancer” with “cervical cancer.” The scale consists of six items, five of which are measured on a four-point Likert scale, and one measured on a five-point Likert scale. The total score ranges from 6 to 25, with a score of 15 or higher indicating a significantly high degree of psychological distress. The secondary outcome was the intention to undergo further examination for cervical cancer, which was determined with a yes/no question.

The covariates measured included demographic characteristics, mental health, perceived health competence, and attitudes toward cervical cancer screening. Mental health was assessed using the Japanese version of the K6 scale, which was designed to measure depression and anxiety [23, 24]. The K6 scale consists of six items rated on a five-point Likert scale. The total score ranges from 0 to 24, with higher scores indicating higher degrees of depression and anxiety. Perceived health competence was measured using a Japanese version of the modified Perceived Health Competence Scale [25]. This tool, which measures self-efficacy regarding general health-related behaviors [26], contains eight items measured on a five-point Likert scale. The total score ranges from 8 to 40, with higher scores indicating higher degrees of self-efficacy. Attitudes toward cervical cancer screening were measured using a modified version of the 16-item Attitude toward Breast Cancer Screening Scale [21]. The items were measured on a five-point Likert scale. In this study, we replaced “breast cancer” in the original scale with “cervical cancer.” This scale includes four factors: barriers toward screening, lack of perceived importance of screening, perceptions of screening, and subjective norms related to screening. The score for each factor was calculated. The total score ranges from 4 to 20, with lower scores indicating better attitudes toward cervical cancer screening. The Japanese versions of the three scales mentioned above have been validated [21, 23, 25].

### Statistical analysis

First, we compared the participants’ baseline characteristics between the intervention and control groups, and then calculated the proportion of psychological distress (i.e., Cancer Worry Scale score ≥ 15) among the participants before and after the intervention in the intervention and control groups by positive and negative screening results. To take into account psychological distress at baseline, the proportion of psychological distress after the intervention was also calculated specifically among participants without psychological distress before the intervention. Finally, we estimated the effect of the intervention with odds ratios (ORs) and 95% confidence intervals (CIs) by positive (each cytological grade) and negative screening results. All analyses were based on intention-to-treat.

## Results

**Fig. 1:**
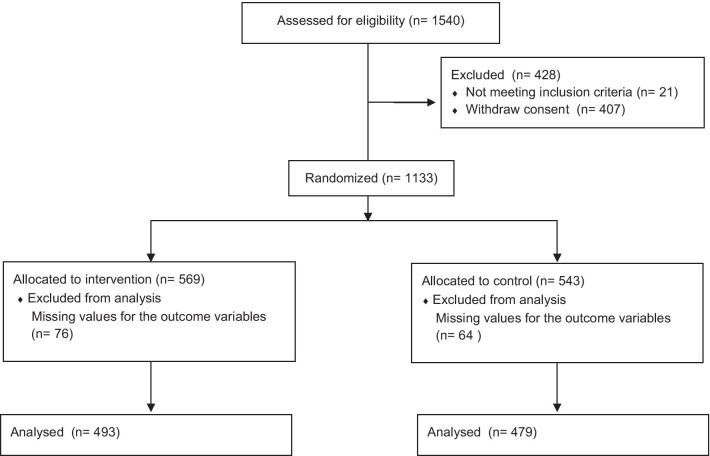
Participant flow from recruitment

Figure 1 shows the flowchart of the participants. Of the 1540 women invited, 1133 participated in the study, 584 in the intervention group and 549 in the control group. We excluded those aged 70 years or older (15 and 6 participants in the intervention and control groups, respectively) and those with missing values for the outcome variables (76 and 64 participants in the intervention and control groups, respectively). Finally, 972 participants were included in the analysis: 493 in the intervention group and 479 in the control group.

Table 1 compares the participants’ characteristics between the intervention and control groups. The mean age of participants was 45 years. Most had graduated from junior college or university (79%), had a spouse/partner (90%), and had experienced childbirth at least once (87%). Regarding occupation, the participants mainly held either part-time jobs (40%) or were housewives (32%), and 25% of the participants had a smoking history. The mean K6 score was 3.1. Most (92%) of the participants had undergone cervical cancer screening in the past, but had mostly received negative results (90%). Only 6% had previously undergone further examination. There were no significant differences between the intervention and control groups with regard to general characteristics, perceived health competence, or attitudes toward screening.

**Table 1:** Comparison of participants’ characteristics

	Intervention group n (%)	Control group n (%)
	493 (50.7)	479 (49.3)
**Age, years**		
Mean	44.6 (10.3)	44.9 (10.1)
20–29	17 (3.5)	15 (3.1)
30–39	153 (31.0)	133 (27.8)
40–49	180 (36.5)	184 (38.4)
50–59	75 (15.2)	75 (15.7)
60–69	58 (11.8)	55 (11.5)
Missing	10 (2.0)	17 (3.6)
**Educational level**		
Junior high school	2 (0.4)	4 (0.8)
High school	82 (16.3)	104 (21.7)
Vocational school/junior college	191 (38.7)	184 (38.4)
College/university	214 (43.4)	183 (38.2)
Missing	4 (0.8)	4 (0.8)
**Marital status**		
Married/partnered	441 (89.5)	428 (89.4)
Widowed/divorced	23 (4.7)	27 (5.6)
Single	28 (5.7)	22 (4.6)
Missing	1 (0.2)	2 (0.4)
**Occupation**		
Public officer	87 (17.7)	73 (16.2)
Self-employed	26 (5.3)	21 (4.4)
Part-time job	189 (38.3)	203 (42.4)
Housewife	161 (32.7)	153 (31.9)
None	7 (1.4)	10 (2.1)
Student	8 (1.6)	5 (1.0)
Other	14 (2.8)	12 (2.5)
Missing	1 (0.2)	2 (0.4)
**Smoker**		
At some point	111 (22.5)	128 (26.7)
Never	380 (77.1)	350 (73.1)
Missing	2 (0.4)	1 (0.2)
**Childbirth experience**		
No	69 (14.0)	60 (87.1)
Yes	423 (85.8)	417 (87.1)
Missing	1 (0.2)	2 (0.4)
**Diagnosed history of cancer**		
Yes	18 (3.7)	11 (2.3)
No	447 (90.7)	455 (95.0)
Missing	28 (5.7)	13 (2.7)
**Mental health (K6)**		
Mean K6 score (SD)	3.2 (3.6)	3.0 (3.6)
Missing	11 (2.2)	8 (1.7)
**History of cervical cancer screening**		
Yes	452 (91.7)	438 (91.4)
No	32 (6.5)	26 (5.4)
Missing	9 (1.8)	15 (3.1)
**History of positive cervical cancer screening result**^a^		
Yes	29 (6.4)	23 (5.3)
No	406 (89.8)	399 (91.1)
Don’t know	0 (0.0)	1 (0.2)
Missing	17 (3.8)	15 (3.4)
**History of further examination**^a^		
Yes	27 (6.0)	22 (5.0)
No	421 (93.1)	410 (93.6)
Missing	4 (0.9)	6 (1.7)
**Perceived health competence**^b^		
Mean (SD)	23.6 (3.0)	23.6 (3.0)
**Attitude toward cervical cancer screening**^b^		
Barriers toward screening	9.8 (3.7)	9.7 (3.4)
Lack of perceived importance of screening	7.0 (2.3)	7.1 (2.2)
Perceptions of screening	13.0 (3.2)	12.7 (3.0)
Subjective norms related to screening	10.6 (3.4)	10.7 (3.3)

*SD* standard deviation

^a^The proportion of women with a history of positive cervical cancer screening results differs from other variables in the number of denominators. Among the women with a history of cervical cancer screening, this is the proportion with experience of positive results

^b^The number is different because the score was calculated by excluding those with missing responses on the questionnaire

As shown in Table 2, the proportion of psychological distress (i.e., Cancer Worry Scale score ≥ 15) at baseline was not significantly different between the intervention and control groups. Among those who were informed of a positive result, this proportion increased more in the control group (from 47 to 79%) than in the intervention group (from 44 to 60%). The same trend was observed for the six positive result patterns. Among those who were informed of a negative screening result, this proportion did not increase in either the intervention or control group.

**Table 2:** Proportion of psychological distress^a^ before and after receiving the hypothetical result in the intervention and control groups by screening results

	Intervention group			Control group		
		Before	After		Before	After
	Number	n (%)	n (%)	Number	n (%)	n (%)
Positive result^b^	423	171 (43.7)	252 (59.6)	407	85 (46.9)	322 (79.1)
ASC-US (Lowest grade)	73	32 (43.8)	49 (67.1)	67	29 (43.3)	52 (77.6)
ASC-H	60	29 (48.3)	33 (55.0)	67	23 (34.3)	55 (82.1)
LSIL	71	32 (45.1)	44 (62.0)	62	32 (51.6)	46 (74.2)
HSIL	77	32 (41.6)	39 (50.7)	66	29 (43.9)	52 (78.8)
SCC (Highest grade)	76	31 (40.8)	46 (60.5)	73	43 (58.9)	54 (74.0)
Not informed of the cytological stages	66	29 (43.9)	41 (62.1)	72	35 (48.6)	63 (87.5)
Negative result	70	31 (44.3)	31 (44.3)	72	34 (47.2)	29 (40.3)

ASC-US, ASC-H, LSIL, HSIL, and SCC are abbreviations of the cytological stages

^a^The total score ranges from 6 to 25, with higher scores indicating a higher degree of psychological distress. Participants with higher psychological distress scores received high cancer worry scores (≥ 15)

^b^All six patterns of positive results

Among those without psychological distress at baseline, the proportion of psychological distress after being informed of a positive screening result appeared to be higher in the control group (64%) than in the intervention group (36%). This was not the case among the participants who were informed of a negative result (10% and 11% in the intervention and control groups, respectively) (data not shown).

After the intervention, among those who were informed of a positive screening result, psychological distress was higher in the control group than in the intervention group (OR: 2.57, 95% CI: 1.87–3.54), but this was not the case among those who were informed of a negative result (OR: 0.85, 95% CI: 0.41–1.74). Among those who were informed of a positive screening result, 95% and 97% of those in the intervention and control groups, respectively, reported that they would undergo further examination.

## Discussion

We evaluated whether the provision of information through a leaflet would help reduce psychological distress without affecting the intention to undergo further examination among women undergoing cervical cancer screening. As a result, psychological distress appeared to be lower in the intervention group (women receiving a leaflet) than in the control group (women not receiving it) among those who received a hypothetical positive screening result at any cytological stage, while women in both groups maintained the intention to undergo further examination. Our findings suggest that, irrespective of cytological grade, the psychological distress associated with a positive cervical cancer screening result can be averted if these women receive the relevant information, which does not affect their intention to undergo further examination.

Generally, a positive screening result is ambiguous and worrisome to women who are not familiar with the process. Therefore, it is necessary to resolve this ambiguity to alleviate their concerns. Reportedly, the provision of simple but thorough information is useful in improving their understanding [27, 28]. Therefore, we clearly described the possibility of false positives, false negatives, and overdiagnosis in a leaflet. We also explicitly stated that a positive screening result is not a cancer diagnosis as well as specifying the benefits of screening.

While the provision of such information will be helpful for women to manage potential psychological distress associated with a positive screening result, it will not interfere with their intention to undergo further examination in case of a positive result. This is clinically important because the ultimate goal of screening is to enable early detection and treatment of the disease, which requires further examination of those who screen positive.

The policy implications of this study are the fact that a simple intervention of providing relevant information using leaflets can reduce cervical cancer-related psychological distress without affecting the intention to undergo further examination. This should be practically and financially feasible; the leaflets simply need to be sent with the letters containing the screening results.

Our study had several limitations. First, since the screening results were hypothetical, it is unclear whether such information provision would reduce psychological distress in actual situations. Yet, even in a hypothetical situation, the increase in psychological distress was greater among those who did not receive information. This result indicates the potential impact of information provision on psychological distress. Second, in the study setting, women might have read the leaflet more carefully than in a routine clinical setting. If this was the case, the alleviating effect of the leaflet on psychological distress might have been overestimated. On the other hand, their intention to undergo further examination was less likely to be affected because the proportion of the study participants who had such an intention was over 90%. According to the national cancer statistics, the proportion of women who actually received a positive result of cervical cancer screening and took further examination was about 80% [7]. Third, we need to be cautious about generalizing the findings because the participants of this study had a high educational level, with 79% having graduated from college/university, whereas only 30% of women nationwide have attained that level of education [29]. Therefore, it is necessary to investigate whether the message in the leaflet would be easily comprehensible to the majority of women undergoing cervical cancer screening.

## Conclusions

In conclusion, information provision would help reduce psychological distress among women who receive a positive cervical cancer screening result without affecting their intention to undergo further examination. We recommend that cervical cancer screening programs provide participants with all relevant information.

## Data Availability

The datasets used and analyzed during the current study are available from the corresponding author upon reasonable request.

## Abbreviations

TBS: The Bethesda System
ORs: Odds ratios
CIs: Confidence intervals
